# Conjugated Linoleic Acid Ameliorates High Fat-Induced Insulin Resistance via Regulating Gut Microbiota–Host Metabolic and Immunomodulatory Interactions

**DOI:** 10.3390/nu16081133

**Published:** 2024-04-11

**Authors:** Linjun Wu, Shijie Ye, Xiangfei Deng, Zhengwei Fu, Jinjun Li, Chunlei Yang

**Affiliations:** 1 College of Biotechnology and Bioengineering, Zhejiang University of Technology, Hangzhou 310014, China; 2112005190@zjut.edu.cn (L.W.); yeshijie0423@163.com (S.Y.); 2112005101@zjut.edu.cn (X.D.); 2 Food Science Institute, Zhejiang Academy of Agricultural Sciences, Hangzhou 310021, China; 3 Key Laboratory of Postharvest Preservation and Processing of Vegetables (Co-Construction by Ministry and Province), Ministry of Agriculture and Rural Affairs, Hangzhou 310021, China; 4 Key Laboratory of Post-Harvest Handling of Fruits, Ministry of Agriculture and Rural Affairs, Hangzhou 310021, China; 5 Key Laboratory of Fruits and Vegetables Postharvest and Processing Technology Research of Zhejiang Province, Hangzhou 310021, China

**Keywords:** conjugated linoleic acid, insulin resistance, immunoregulation, gut microbiota, microbial metabolites

## Abstract

Interaction between gut microbiota, host immunity and metabolism has been suggested to crucially affect the development of insulin resistance (IR). This study aims to investigate how gut microbiota, inflammatory responses and metabolism in individuals with IR are affected by the supplementation of conjugated linoleic acid (CLA) and how this subsequently affects the pathophysiology of IR by using a high-fat diet-induced IR mouse model. Serum biochemical indices showed that 400 mg/kg body weight of CLA effectively attenuated hyperglycemia, hyperlipidemia, glucose intolerance and IR, while also promoting antioxidant capacities. Histomorphology, gene and protein expression analysis revealed that CLA reduced fat deposition and inflammation, and enhanced fatty acid oxidation, insulin signaling and glucose transport in adipose tissue or liver. Hepatic transcriptome analysis confirmed that CLA inhibited inflammatory signaling pathways and promoted insulin, PI3K-Akt and AMPK signaling pathways, as well as linoleic acid, arachidonic acid, arginine and proline metabolism. Gut microbiome analysis further revealed that these effects were highly associated with the enriched bacteria that showed positive correlation with the production of short-chain fatty acids (SCFAs), as well as the improved SCFAs production simultaneously. This study highlights the therapeutic actions of CLA on ameliorating IR via regulating microbiota–host metabolic and immunomodulatory interactions, which have important implications for IR control.

## 1. Introduction

The global obesity epidemic is estimated to have more than tripled in the last two decades. Obesity is a critical risk factor that is associated with various metabolic disorders including insulin resistance (IR) [1]. IR is a clinical state in which the glucose uptake capacity of tissues, such as the liver and adipose tissue, is reduced because of the decreased biological effect of insulin compared to healthy individuals, and is considered to be a major pathological mechanism related to the progression of type 2 diabetes [2]. The mechanisms underlying IR are multifactorial and include ectopic lipid accumulation as well as systemic chronic inflammation [3,4]. Therefore, successful strategies for the prevention and treatment of obesity-induced IR should synergistically provide metabolic and immunological benefits. 

The critical role of gut microbiota and its metabolite in affecting host metabolic and immune function has been extensively reported [5]. Obesity-related problems and the onset of metabolic complications originate, at least in part, from gut bacterial dysbiosis [6]. For example, high-fat (HF) feeding has been indicated to change gut microbiota and environment through ways such as decreasing gut bacterial diversity, suppressing favorable bacteria, shifting the ratio of Firmicutes to Bacteroides, as well as altering short-chain fatty acids (SCFAs) profile [7]. These alterations can reshape the immune environment of the gut, leading to changes in intestinal permeability and immune cytokines trafficking, which affect systemic inflammation and IR [8]. Therefore, the gut microbiota is widely recognized as an important therapeutic target to control obesity-related IR.

Conjugated linoleic acid (CLA) is a mixture of geometrical and positional isomers of linoleic acid that mainly present in dairy products and the meat of ruminants, and can also be endogenously synthesized in non-ruminant animals and humans [9]. Of the various isomers, *cis*-9, *trans*-11-CLA and *trans*-10, *cis*-12-CLA are the most utilized, and numerous biological effects have been reported in associate with their interactions, such as the properties of anti-obesity and anti-inflammation [10]. It is worth noting that while the potential effects of CLA on regulating adipocyte function, lipid metabolism, and insulin sensitivity in humans have been proposed, the regulatory effects remain controversial due to the fact that reports from clinical trials of the mixed isomers are sometimes conflicting [11,12,13,14,15,16,17]. A previous study also showed that the effect of CLA on ameliorating hepatic fat accumulation and inflammation was more suitable for leptin-deficient ob/ob mice than wild-type mice, and this effect was correlated with the modulation of gut microbiota [18]. However, data on the underlying effects of CLA mixtures on the mitigation of obesity-induced IR via modulating the crosstalk between gut microbiota and host metabolism, as well as immune function, are still missing. 

Therefore, to investigate whether the administration of CLA can ameliorate obesity-induced IR through regulating metabolic and immunomodulatory signaling, as well as to elucidate whether the modulation was derived from the function of CLA on gut microbiome, we analyzed the changes in glucose tolerance and homeostasis, insulin sensitivity, lipid profile, inflammatory responses, colonic microbial community and metabolites in C57BL/6J mice induced with IR by being fed a HF diet, comparing those administered with CLA to those without.

## 2. Materials and Methods

### 2.1. Animal Experiment

All animal experiments were conducted in accordance with the guidelines approved by the animal ethics committee of Zhejiang Academy of Agricultural Sciences (protocol number: 2022ZAASLA89). Male C57BL/6J mice (six-week-old) were randomly assigned to one of the four diet groups (*n* = 6 for each group) and fed for 15 weeks under standard conditions containing a 12/12 h light/dark cycle: the control diet (NC group, 11.7% calories from fat, MD17121, Medicience, Ltd., Yangzhou, China); the control diet with 400 mg/kg body weight CLA supplemented every day (NC+CLA group); the high-fat diet (HF group, 45.0% calories from fat, MD12032, Medicience, Ltd., Yangzhou, China); the high-fat diet with 400 mg/kg body weight CLA supplemented every day (HF+CLA group). The CLA product used was received from Qingdao Auhai biotech Co., Ltd. (Qingdao, China) with the fatty acid composition of 80.4% total CLA. After the treatments, mice were sacrificed and their adipose, liver and colon tissues as well as colonic contents were carefully collected and stored at −80 °C for further analysis.

To evaluate the effect of CLA on insulin signaling, a second set of mice (six-week-old) were randomly assigned to one of the four groups: NC, NC+CLA, HF and HF+CLA (*n* = 4 for each group), and insulin (6 U/kg body weight) was intraperitoneally injected 10 min before the sacrifice at the end of the 15 weeks [19]. Then, their adipose and liver tissues were collected for further analysis.

### 2.2. Glucose and Insulin Tolerance Test

During the last week of intervention, a glucose tolerance test (GTT) and insulin tolerance test (ITT) were performed. For GTT, animals were intraperitoneally injected with glucose (2 g/kg body weight) after overnight fasting [19]. For ITT, animals were intraperitoneally injected with insulin (0.75 U/kg body weight) after fasting for 6 h [20]. Blood samples were collected from tail veins and blood glucose levels were immediately measured before injection (t = 0 min) and at selected time points (30, 60, 90 and 120 min) after injection using FreeStyle Optium Neo (Abbott, Chicago, IL, USA).

### 2.3. Biochemical Analyses

The levels of serum glucose, fasting insulin (FINS), triglyceride (TG), total cholesterol (TC), leptin, non-esterified fatty acids (NEFA) and adiponectin (ADPN), activities of serum aspartate aminotransferase (AST) and alanine aminotransferase (ALT), hepatic superoxide dismutase (SOD), catalase (CAT) and glutathione peroxidase (GSH-Px), colonic glucagon-like peptide-1 (GLP-1) were measured using the commercial enzyme-linked immunosorbent assay (ELISA) kits (Nanjing Jiancheng Bioengineering Institute, Nanjing, China; Jiangsu Jingmei Biotechnology Co., Ltd., Yancheng, China) according to the protocol of manufacturers. The homoeostatic model assessment of insulin resistance (HOMA-IR) was calculated according to the formula of fasting insulin × fasting glucose/22.5 [21].

### 2.4. Analysis of Histology and Immunofluorescence Staining

The epididymal adipose tissue and liver tissue were collected, respectively, and fixed with 4% paraformaldehyde. After paraffin embedding and slicing, the samples were either stained using hematoxylin-eosin (H&E), or anti-F4/80 (1:1000, GB11027, Servicebio, Wuhan, China) antibody using immunofluorescence staining, respectively [22]. Immunofluorescence staining was also conducted for the samples of adipose tissue using an anti-GLUT-4 (1:1000, GB11244, Servicebio, Wuhan, China) antibody. The average adipocyte size and lipid droplet area were determined using ImageJ software (v2.1.0/1.53c). 

### 2.5. Gut Microbiota and SCFAs Analysis

Colonic microbiota analysis was performed by 16S rRNA sequencing using NovaSeq6000 platform (Beijing Novogene Biological Information Technology Co., Ltd., Beijing, China). Sequencing amplicon was prepared by amplifying V3-V4 region of the bacterial 16S rRNA gene [23]. QIIME2 (v2022.2) was used to assign the cleaned sequences into amplicon sequence variants (ASV), and the taxonomic assignment of each ASV was performed basing on Silva 138.1 database [24]. SCFAs levels of the colonic content were then detected by the gas chromatography-mass spectrometry (Agilent Technologies, Santa Clara, CA, USA) using the method as reported previously [25]. The 16S rRNA data were deposited in the Sequence Read Archive (SRA; https://www.ncbi.nlm.nih.gov/sra) (accessed on 8 March 2023) under the accession number of PRJNA940316.

### 2.6. Analysis of the Hepatic Transcriptome Profiling

Paired-end sequencing (150 bp) was conducted on Illumina HiSeq4000 platform (Beijing Novogene Biological Information Technology Co., Ltd., Beijing, China). Cleaned data were mapped to the reference genome and reference gene annotations of *Mus musculus* using HISAT2 (v2.2.1), respectively [26]. Gene expression levels were calculated according to fragments per kilobase of transcript per million fragments mapped (FPKM) and the differentially expressed genes (DEGs) among all the groups were assessed using DESeq2 (v3.15) [27]. Benjamini and Hochberg’s procedure was utilized to adjust the *p*-value into a false discovery rate. A threshold of *p* < 0.05 and fold change > 2 was utilized to define DEGs. The functional analysis of these DEGs was conducted by KOBAS (v3.0) [28] to analyze the statistical enrichment of Kyoto Encyclopedia of Genes and Genomes (KEGG) pathways. Protein–protein interaction network for DEG was determined using STRING v10.5 database, and hub genes (degree > 5) were analyzed by Cytoscape (v3.9.1) [29]. The RNA-Seq data were deposited in the Sequence Read Archive (SRA; https://www.ncbi.nlm.nih.gov/sra) (accessed on 24 February 2023) under the accession number of PRJNA938011.

### 2.7. Quantitative Real-Time PCR (qPCR)

After RNA extraction and transformation into cDNA, qPCR was performed in Roche LightCycler 480 System (Roche, Bethesda, MD, USA) with SYBR green, as previously described [30]. The data obtained were finally normalized to the expression of housekeeping gene β-actin using the 2^−∆∆Ct^ method. The specific primers utilized were designed by the Basic Local Alignment Search Tool [BLAST; National Center for Biotechnology Information (NCBI), Bethesda, MD, USA] and presented in Table S1.

### 2.8. Western Blot

To obtain the protein lysates, tissues (*n* = 4 for each group) were homogenized and sonicated using the radio-immunoprecipitation assay buffer (Millipore, Billerica, MA, USA) with phosphatase inhibitors and protease (Roche Diagnostics, Basel, Switzerland) added. Then, immunoblots were conducted as described before [30]. The primary antibodies utilized were anti-AKT (1:1000, CST, #9272, Danvers, MA, USA), anti-phospho-AKT (Ser473) (1:2000, CST, #4060), anti-IκB alpha (1:5000, Abcam, ab32518, Cambridge, UK), anti-IκB alpha (phospho S36) (1:5000, Abcam, ab133462), anti-SAPK/JNK (1:1000, CST, #9252), anti-phospho-SAPK/JNK (Thr183/Tyr185) (1:1000, CST, #9251), anti-GLUT4 (1F8) (1:1000, CST, #2213), anti-GLUT2/SLC2A2 (1:1000, ABclonal, #A9843, Wuhan, China) and anti-β-actin (1:1000, CST, #4967).

### 2.9. Statistical Analysis

Statistical analysis and visualization were performed by SPSS (v22.0) and GraphPad Prism (v9.3.0). Student’s *t*-test was used to compare the differences between the means of two groups. The ANOVA, followed by Tukey’s post hoc test, was utilized to compare the differences of the means among more than 2 groups. *p*-value < 0.05 was considered as statistically significant. Analysis of similarities (ANOSIM) was performed with the “anosim” function in ”vegan” package. Correlations among the bacterial abundances, SCFAs concentrations, physiological and biochemical indicators, as well as gene and protein expressions were determined by Spearman rank correlation coefficient analysis using the “psych” package in R.

## 3. Results

### 3.1. CLA Attenuated Hyperglycemia, Hyperlipidemia and IR in HF-Fed Mice

**Figure 1:**
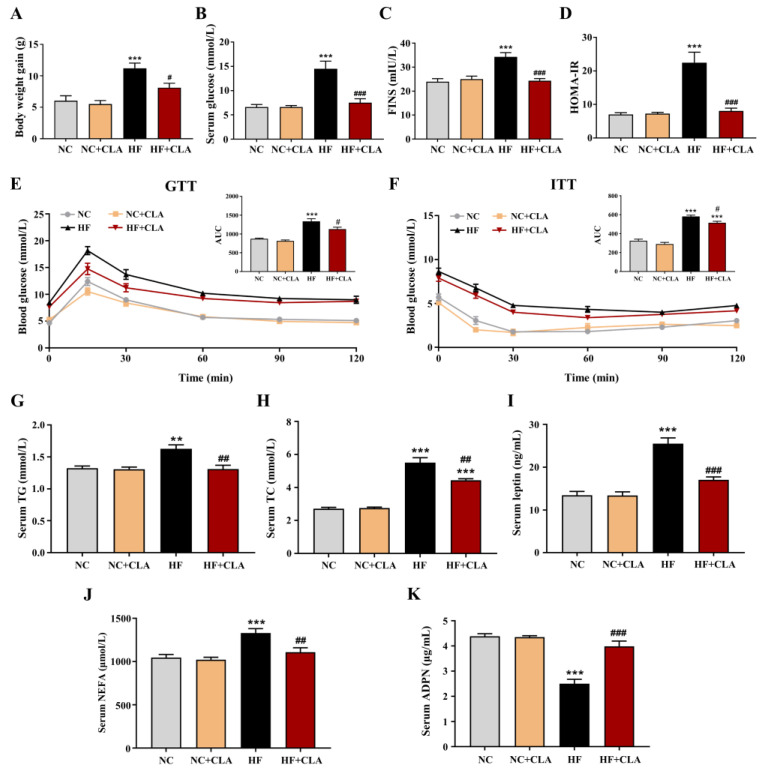
Effects of conjugated linoleic acid (CLA) on the body weight gain (**A**), fasting serum glucose (**B**), fasting serum insulin (FINS) (**C**), homoeostatic model assessment of insulin resistance (HOMA-IR) (**D**), glucose tolerance evaluated by glucose tolerance test (GTT) (**E**), insulin sensitivity evaluated by insulin tolerance test (ITT) (**F**), serum triglyceride (TG) level (**G**), serum total cholesterol (TC) level (**H**), serum leptin level (**I**), serum non-esterified fatty acids (NEFA) level (**J**) and serum adiponectin (ADPN) level (**K**) of mice fed a high-fat (HF) diet. AUC, area under the curve; NC, mice fed control diet for 15 weeks; NC+CLA, mice fed control diet with 400 mg/kg body weight CLA supplemented every day for 15 weeks; HF, mice fed HF diet for 15 weeks; HF+CLA, mice fed HF diet with 400 mg/kg body weight CLA supplemented every day for 15 weeks. Values are presented as mean ± SEM. ** *p* < 0.01, *** *p* < 0.001 vs. NC; ^#^ *p* < 0.05, ^##^ *p* < 0.01, ^###^ *p* < 0.001 vs. HF.

The C57BL/6J mice fed a HF diet had a higher body weight gain compared with NC group, whereas CLA supplementation effectively reduced the body weight gain of HF mice (Figure 1A, *p* < 0.05). Results from the levels of fasting glucose and FINS, HOMA-IR, GTT and ITT demonstrated that the HF feeding led to an obvious hyperglycemia as well as an impaired glucose tolerance and insulin sensitivity, whereas the supplementation of CLA significantly reduced the fasting glucose and insulin levels, increased glucose tolerance and insulin sensitivity, thereby relieving the IR status in HF mice (Figure 1B–F, *p* < 0.05). Regarding lipid metabolism, the results revealed that CLA supplementation significantly lowered the levels of serum TG, TC, leptin and NEFA, and significantly enhanced the level of serum ADPN in HF mice (Figure 1G–K, *p* < 0.05).

### 3.2. CLA Alleviated Adipocyte Hypertrophy, Metabolic Disorder, Inflammation and IR in HF-Fed Mice

**Figure 2:**
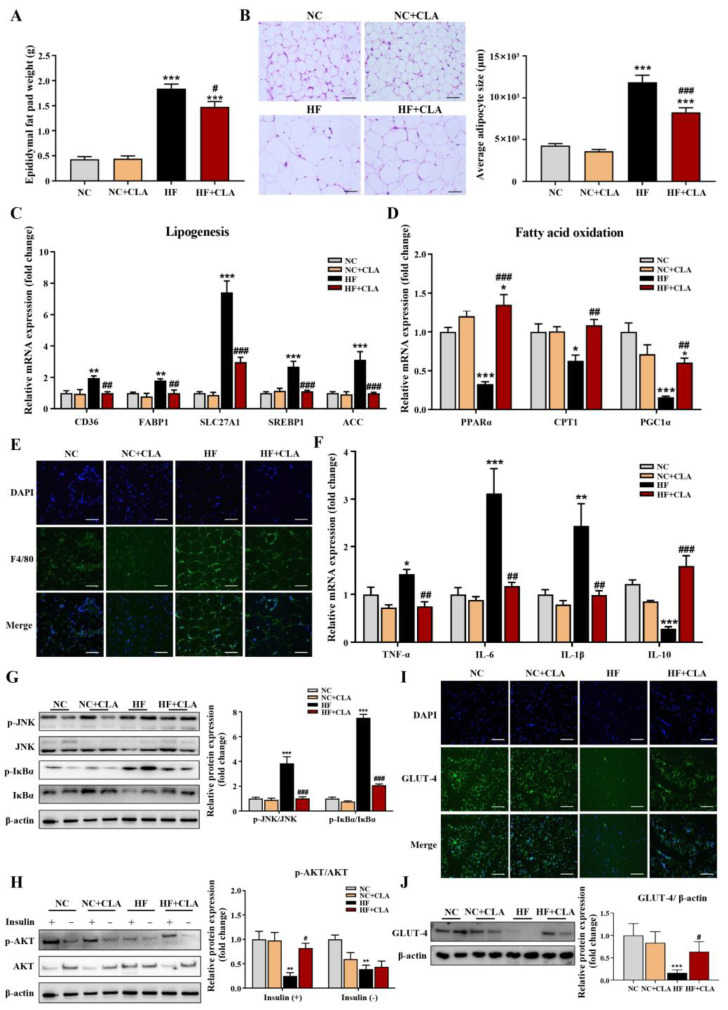
Effects of conjugated linoleic acid (CLA) on the histology, lipid metabolism, inflammation and insulin resistance of adipose tissue in high-fat (HF) feeding mice. (**A**) Epididymal fat pad weight. (**B**) Histological images of H&E-stained adipose tissue and average adipocyte size, scale bar: 100 µm. (**C**) Relative mRNA expressions of lipogenesis-related genes *CD36*, *FABP1*, *SLC27A1*, *SREBP1* and *ACC*. (**D**) Relative mRNA expressions of fatty acid oxidation-related genes *PPARα*, *CPT1* and *PGC1α*. (**E**) Immunofluorescence staining of F4/80 (green), nuclei were stained with DAPI (blue), scale bar: 100 µm. (**F**) Relative mRNA expressions of *TNF-α*, *IL-6*, *IL-1β*, and *IL-10*. (**G**) Protein expression of phosphorylated (p)-JNK/JNK and p-IκBα/IκBα. (**H**) Protein expression of p-AKT/AKT. (**I**) Immunofluorescence staining of GLUT-4 (green), nuclei were stained with DAPI (blue), scale bar: 100 µm. (**J**) Protein expression of GLUT-4/β-actin. β-actin was used as reference protein. NC, mice fed control diet for 15 weeks; NC+CLA, mice fed control diet with 400 mg/kg body weight CLA supplemented every day for 15 weeks; HF, mice fed HF diet for 15 weeks; HF+CLA, mice fed HF diet with 400 mg/kg body weight CLA supplemented every day for 15 weeks. Values are presented as mean ± SEM. * *p* < 0.05, ** *p* < 0.01, *** *p* < 0.001 vs. NC; ^#^ *p* < 0.05, ^##^ *p* < 0.01, ^###^ *p* < 0.001 vs. HF.

According to Figure 2A,B, HF feeding significantly increased the epididymal fat pad weight and average adipocytes size of adipose tissue compared with the NC group, but the CLA supplementation significantly lowered these increases in HF mice (*p* < 0.05). Results from the expression of genes related to lipid metabolism further demonstrated that the HF+CLA mice had significantly reduced expression levels of genes related to lipogenesis, including *CD36*, *FABP1*, *SLC27A1*, *SREBP1* and *ACC*, and a significantly increased expression of genes involved in fatty acid oxidation, including *PPARα*, *CPT1* and *PGC1α*, compared with HF mice (Figure 2C,D, *p* < 0.05). Regarding inflammatory responses, the immunofluorescence staining results revealed that the adipose tissue of HF mice had an obviously enhanced expression of the proinflammatory macrophage activation marker F4/80 compared with NC mice, but CLA supplementation effectively reduced the increased expression of F4/80 in HF mice (Figure 2E). Correspondingly, the gene expressions of proinflammatory cytokine *TNF-α*, *IL-6* and *IL-1β* and the protein expressions of phosphorylated (p)-JNK/JNK and p-IκBα/IκBα in adipose tissue, which are key inflammatory signaling regulators, were also significantly reduced in HF mice with the supplementation of CLA (Figure 2F,G, *p* < 0.05). In contrast, the gene expression of anti-inflammatory cytokine *IL-10* in the adipose tissue of HF mice was significantly increased with the CLA supplementation compared with the HF mice (Figure 2F, *p* < 0.05). In consistency with these alterations in lipid metabolism and inflammation, the insulin-induced expression of p-AKT/AKT (Figure 2H) and glucose transporter GLUT-4 (Figure 2I,J) were both substantially enhanced in the adipose tissue of HF+CLA mice compared with HF mice (*p* < 0.05), which had been reduced in the HF group compared with the NC group.

### 3.3. CLA Altered Hepatic Lipid Accumulation, Gene Expression Profile and IR-Related Signaling in HF-Fed Mice

**Figure 3:**
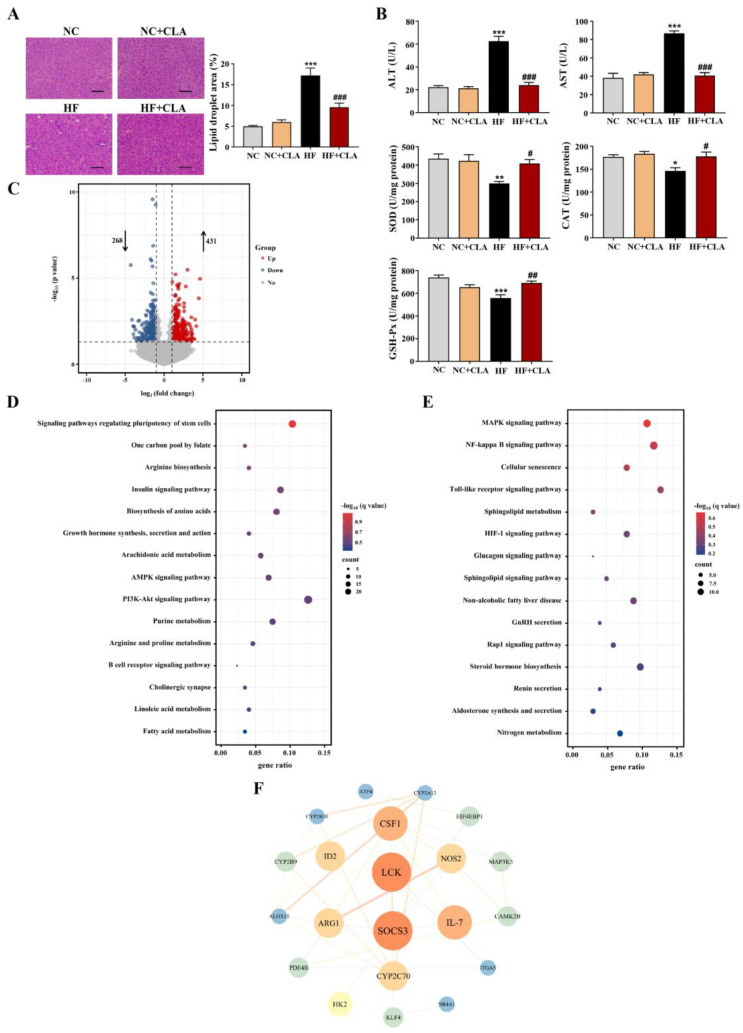
Effects of conjugated linoleic acid (CLA) on the histology, antioxidant function and transcriptome profile of liver in high-fat (HF) feeding mice. (**A**) Histological images of H&E-stained hepatic tissue and the percentage of the hepatic lipid droplet area, scale bar: 100 µm. (**B**) Activities of serum alanine aminotransferase (ALT) and aspartate aminotransferase (AST), as well as hepatic superoxide dismutase (SOD), catalase (CAT) and glutathione peroxidase (GSH-Px). (**C**) Volcano plot of the differentially expressed genes (DEGs) (*p* < 0.05, fold change > 2) based on the comparison between the HF+CLA and HF groups; the dot lines indicate the significance threshold, the blue circle indicates the genes that were significantly downregulated in the HF+CLA group compared with the HF group, and the red circle indicates the genes that were significantly upregulated in the HF+CLA group compared with the HF group. (**D**) KEGG pathways of the DEGs that were upregulated in the HF+CLA group compared with the HF group (only the top 15 pathways were shown). (**E**) KEGG pathways of the DEGs that were downregulated in the HF+CLA group compared with the HF group (only the top 15 pathways were shown). (**F**) Protein–protein interaction (PPI) analysis of the DEGs between the HF+CLA and HF groups; the node color indicates the clustering coefficient (from low to high values: blue to red), the node size is proportional to the number of degrees, and the edge indicates the interaction (a thicker edge indicates a lower betweenness). NC, mice fed control diet for 15 weeks; NC+CLA, mice fed control diet with 400 mg/kg body weight CLA supplemented every day for 15 weeks; HF, mice fed HF diet for 15 weeks; HF+CLA, mice fed HF diet with 400 mg/kg body weight CLA supplemented every day for 15 weeks. Values are presented as mean ± SEM. * *p* < 0.05, ** *p* < 0.01, *** *p* < 0.001 vs. NC; ^#^ *p* < 0.05, ^##^ *p* < 0.01, ^###^ *p* < 0.001 vs. HF.

**Figure 4:**
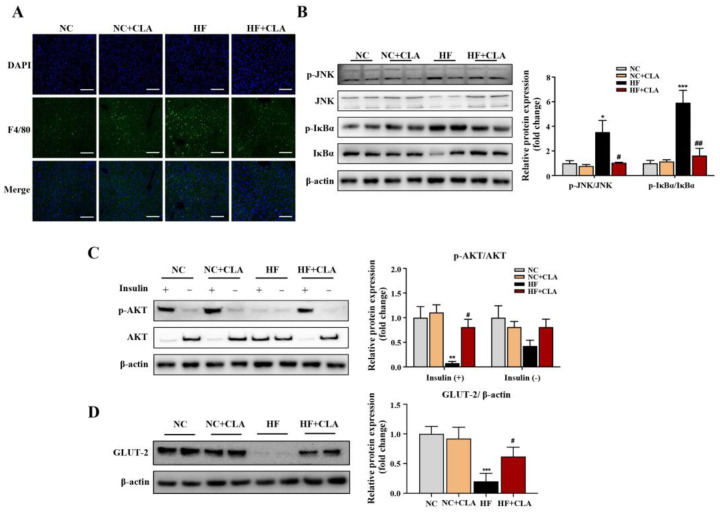
Effects of conjugated linoleic acid (CLA) on the inflammatory responses and insulin resistance of the liver in high-fat (HF) feeding mice. (**A**) Immunofluorescence staining of F4/80 (green), nuclei were stained with DAPI (blue), scale bar: 100 µm. (**B**) Protein expression of phosphorylated (p)-JNK/JNK and p-IκBα/IκBα. (**C**) Protein expression of p-AKT/AKT. (**D**) Protein expression of GLUT-2/β-actin. β-actin was used as reference protein. NC, mice fed control diet for 15 weeks; NC+CLA, mice fed control diet with 400 mg/kg body weight CLA supplemented every day for 15 weeks; HF, mice fed HF diet for 15 weeks; HF+CLA, mice fed HF diet with 400 mg/kg body weight CLA supplemented every day for 15 weeks. Values are presented as mean ± SEM. * *p* < 0.05, ** *p* < 0.01, *** *p* < 0.001 vs. NC; ^#^ *p* < 0.05, ^##^ *p* < 0.01 vs. HF.

According to the H&E staining results, the HF mice exhibited a marked increase in the lipid droplets area in the liver compared with the NC mice, whereas the supplementation of CLA effectively decreased the lipid droplets accumulation (Figure 3A, *p* < 0.05). In line with these histological observations, impaired hepatic function and antioxidant capacity were also observed in HF mice with significantly increased ALT and AST activities, and reduced SOD, CAT and GSH-Px activities detected (HF vs. NC). However, the supplementation of CLA alleviated HF-induced hepatic damage; the HF+CLA mice showed significantly reduced ALT and AST activities but increased SOD, CAT and GSH-Px activities compared with the HF mice (Figure 3B, *p* < 0.05). The liver transcriptome results further demonstrated that the CLA supplementation substantially modified the hepatic transcriptional profile of the mice fed a HF diet (Figure S1). Based on the comparison of the DEGs among different groups, there were 431 genes upregulated and 268 genes downregulated in the HF+CLA group compared with the HF group (Figure 3C, *p* < 0.05, fold change > 2). According to the functional analysis of these DEGs, there were 36 pathways significantly enriched from the upregulated DEGs in the HF+CLA group compared with the HF group, including the insulin signaling pathway, PI3K-Akt signaling pathway, AMPK signaling pathway, arachidonic acid metabolism, linoleic acid metabolism, purine metabolism, arginine and proline metabolism (Figure 3D, *p* < 0.05). For the downregulated DEGs in the HF+CLA mice compared with the HF mice, there were 18 pathways significantly enriched, including the MAPK signaling pathway, NF-kappa B signaling pathway, Toll-like receptor signaling pathway, HIF-1 signaling pathway and non-alcoholic fatty liver disease (Figure 3E, *p* < 0.05). Moreover, the immunofluorescence staining and Western blot results certified that the expression level of the proinflammatory macrophage activation marker F4/80 (Figure 4A), as well as the p-JNK/JNK and p-IκBα/IκBα in the liver of the HF mice, were all substantially reduced by CLA supplementation (Figure 4B, *p* < 0.05). Meanwhile, the insulin-induced hepatic expression of p-AKT/AKT (Figure 4C) and GLUT-2 (Figure 4D) were both substantially enhanced in the HF+CLA mice compared with HF mice (*p* < 0.05). Protein–protein interaction (PPI) analysis results further certified the close interaction between the hub genes (degree > 5) involved in inflammation (*LCK*, *MAP3K5*, *CAMK2B*), metabolism (*ARG1*, *NOS2*, *CYP2C70*, *CYP2B9*, *CYP2B10*, *CYP3A13*, *ALOX15*, *PDE4B*) and insulin signaling (*HK2*, *SOCS3*, *NR4A1*, *ITGA5*, *ATF4*, *CSF1*, *EIF4EBP1*) (Figure 3F). 

### 3.4. CLA Altered the Gut Microbiota in HF-Fed Mice

**Figure 5:**
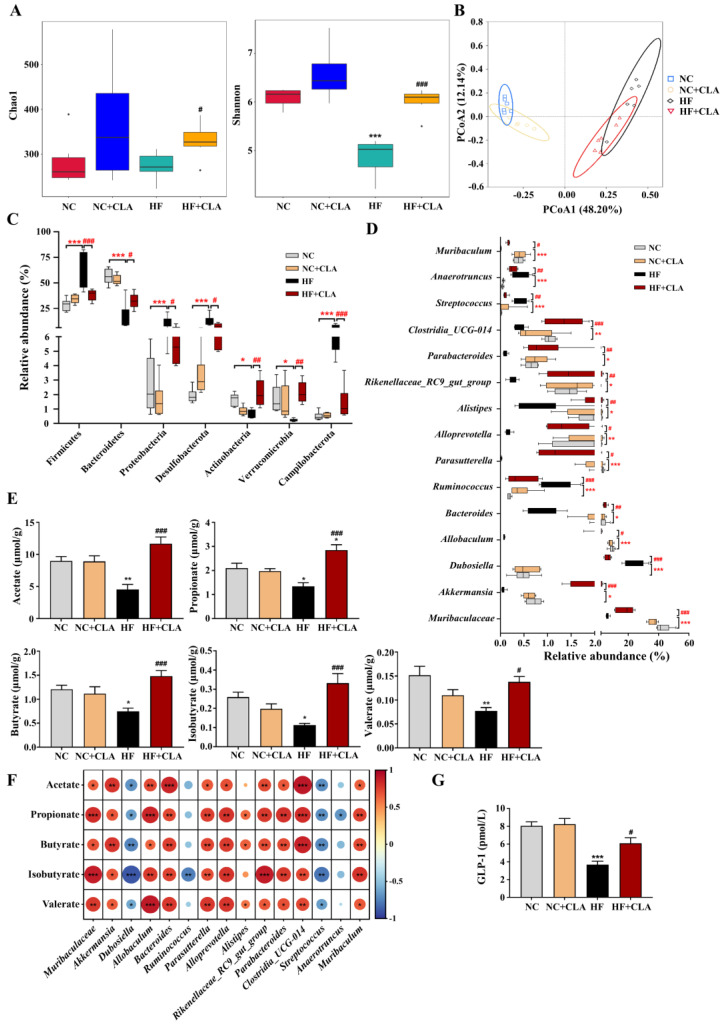
Effects of conjugated linoleic acid (CLA) on the colonic microbial community and metabolites of high-fat (HF) feeding mice. (**A**) Colonic bacterial alpha diversity based on Chao1 and Shannon indexes. (**B**) Principal coordinate analysis (PCoA) of colonic bacterial diversity basing on the Bray–Curtis. (**C**) Dominant phyla of the colonic bacteria with a relative abundance > 0.1% in all groups. (**D**) Dominant genera of the colonic bacteria with a relative abundance > 0.1% in all groups. (**E**) Colonic concentrations of acetate, propionate, butyrate, isobutyrate and valerate. (**F**) Correlations between the changes of bacterial abundance and the concentrations of acetate, propionate, butyrate, isobutyrate and valerate using Spearman correlation coefficient analysis; red indicates positive correlation, blue indicates negative correlation, * *p* < 0.05, ** *p* < 0.01, *** *p* < 0.001. (**G**) Colonic concentration of GLP-1. NC, mice fed control diet for 15 weeks; NC+CLA, mice fed control diet with 400 mg/kg body weight CLA supplemented every day for 15 weeks; HF, mice fed HF diet for 15 weeks; HF+CLA, mice fed HF diet with 400 mg/kg body weight CLA supplemented every day for 15 weeks. * *p* < 0.05, ** *p* < 0.01, *** *p* < 0.001 vs. NC; ^#^ *p* < 0.05, ^##^ *p* < 0.01, ^###^ *p* < 0.001 vs. HF (**A**,**C**–**E**,**G**).

In comparison with the NC group, the HF mice had substantially lower bacterial alpha diversity but the supplementation of CLA significantly improved the bacterial richness and evenness in HF mice, as indicated by Chao1 and Shannon indexes (Figure 5A, *p* < 0.05). Principal coordinate analysis (PCoA) according to the Bray–Curtis metric revealed that the supplementation of CLA caused a distinct shift in the bacterial structure profile of the HF mice (Figure 5B), with significant dissimilarities and segregations in the bacterial composition of the NC, HF and HF+CLA groups observed (Table S2). At the phylum level, a significant shift was detected in 7 phyla of the HF mice with CLA supplemented or not. The HF+CLA mice had significantly higher abundances of Bacteroidetes, Actinobacteria and Verrucomicrobia, whereas significantly lower abundances of Firmicutes, Proteobacteria, Desulfobacterota and Campilobacterota in the colon than the HF mice (Figure 5C, *p* < 0.05). At the genus level, HF feeding significantly decreased the relative abundances of *Muribaculaceae*, *Akkermansia*, *Allobaculum*, *Bacteroides*, *Parasutterella*, *Alloprevotella*, *Alistipes*, *Parabacteroides*, *Rikenellaceae_RC9_gut_group*, *Clostridia_UCG-014* and *Muribaculum* in the colon of the mice, whereas the supplementation of CLA significantly upregulated the abundances of these genera in the HF mice (Figure 5D, *p* < 0.05). On the other hand, HF feeding significantly increased the relative abundance of *Dubosiella*, *Ruminococcus*, *Streptococcus* and *Anaerotruncus* in the colon of the mice, whereas the CLA supplementation significantly decreased the abundances of these genera in the HF mice (Figure 5D, *p* < 0.05).

### 3.5. CLA Modulated the Microbiota-Derived SCFAs Production in HF-Fed Mice

The results demonstrated that the productions of acetate, propionate, butyrate, isobutyrate and valerate were significantly decreased in the colon of the HF mice compared with the NC mice, whereas the supplementation of CLA significantly enhanced the productions of these specific SCFAs in the colon of the HF mice (Figure 5E, *p* < 0.05). The Spearman rank correlation analysis of the associations between the gut microbiota and SCFAs further certified that the alterations in colonic microbiota were alined with the alterations in SCFAs productions (Figure 5F), in which the concentrations of acetate, propionate, butyrate, isobutyrate and valerate were significantly positively correlated with the abundances of *Muribaculaceae*, *Akkermansia*, *Allobaculum*, *Bacteroides*, *Parasutterella*, *Alloprevotella*, *Parabacteroides*, *Rikenellaceae_RC9_gut_group*, *Clostridia_UCG-014* and *Muribaculum* (*p* < 0.05), but negatively correlated with the abundances of *Dubosiella* and *Streptococcus*, respectively (*p* < 0.05). Moreover, the concentrations of propionate, butyrate and valerate had significantly positive correlation with the abundance of *Alistipes*, respectively (*p* < 0.05), while propionate and isobutyrate had negative correlation with the abundances of *Anaerotruncus* and *Ruminococcus*, respectively (*p* < 0.05). SCFA production was proposed to induce the intestinal secretion of GLP-1; we verified that the CLA supplementation significantly enhanced the GLP-1 production in the colon of the HF mice (Figure 5G, *p* < 0.05).

### 3.6. Gut Microbiome Orchestrates CLA-Induced IR Restoration

**Figure 6:**
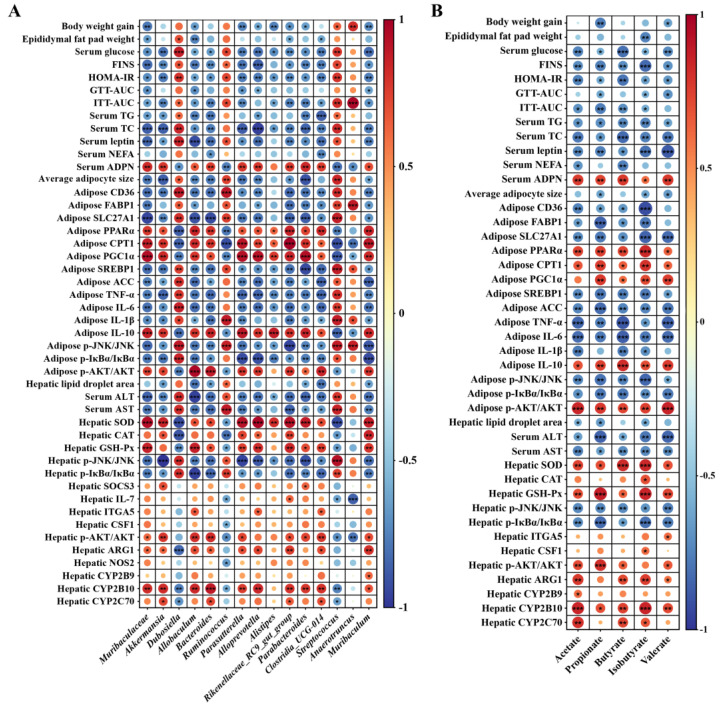
Correlations of the changes of bacterial abundance (**A**), the concentrations of acetate, propionate, butyrate, isobutyrate and valerate (**B**) with the physiological and biochemical parameters, as well as the expression levels of genes and proteins that relate to glucose and lipid homeostasis, glucose tolerance, metabolism, inflammation and insulin resistance using Spearman correlation coefficient analysis; red indicates positive correlation, blue indicates negative correlation. * *p* < 0.05, ** *p* < 0.01, *** *p* < 0.001.

Spearman rank correlation analysis was conducted to investigate the relationship between the gut microbiota or metabolites with the host physiology, metabolism, inflammation, as well as IR, respectively. According to Figure 6A,B, the body weight gain, epididymal fat pad weight, average adipocytes size, hepatic lipid droplet aera, biochemical markers related to hyperglycemia and hyperlipidemia including serum glucose, TG, TC, leptin and NEFA, IR-related indices including FINS, HOMA-IR, GTT-AUC and ITT-AUC, the expression of genes involved in lipogenesis, including *CD36*, *FABP1*, *SLC27A1*, *SREBP1* and *ACC*, as well as involved in inflammation, including *TNF-α*, *IL-1β* and *IL-6*, the phosphorylation of inflammatory regulators including JNK and IκBα, and hepatic function impairment indices including ALT and AST showed significantly negative correlation with the abundance of at least one beneficial bacteria including *Muribaculaceae*, *Akkermansia*, *Allobaculum*, *Bacteroides*, *Parasutterella*, *Alloprevotella*, *Alistipes*, *Rikenellaceae_RC9_gut_group*, *Parabacteroides*, *Clostridia_UCG-014* and *Muribaculum*, as well as at least one specific SCFA including acetate, propionate, butyrate, isobutyrate and valerate, respectively (*p* < 0.05). However, they showed positive correlation with the relative abundance of at least one gut dysbiosis-related bacteria including *Dubosiella*, *Ruminococcus*, *Streptococcus* and *Anaerotruncus*, respectively (*p* < 0.05). In contrast, the activity of anti-oxidative enzymes including SOD, CAT and GSH-Px, the expression level of the genes related to insulin signaling including *SOCS3*, *ITGA5* and *CSF1*, relate to metabolism including *ARG1*, *NOS2*, *CYP2B9*, *CYP2B10* and *CYP2C70*, relate to fatty acid oxidation including *PPARα*, *CPT1* and *PGC1α*, as well as the phosphorylation of AKT showed significantly positive correlation with the abundance of at least one beneficial bacteria or at least one specific SCFA as mentioned above, respectively (*p* < 0.05), but showed negative correlation with the abundance of at least one gut dysbiosis-related bacteria as mentioned above, respectively (*p* < 0.05).

## 4. Discussion

Numerous pieces of evidence indicate that the chronic inflammatory response and activation of the immune system play vital roles in the pathological process of obesity-induced IR [2,3]. As insulin-sensitive tissues, adipose tissue and liver are substantially influenced by obesity at both biomolecular and functional levels [31]. In the present study, we noted that the infiltration of proinflammatory F4/80^+^ macrophages and the expression of genes as well as the proteins related to inflammation were both reduced, whereas the expression of glucose transporter (GLUT) and phosphorylation of AKT, which are key regulators of insulin signaling involved in the suppression of IR [32], were substantially enhanced in the adipose and liver tissues of the HF mice with the supplementation of CLA. This is consistent with the report that anti-inflammatory interventions provide promising therapeutic potential of IR by affecting the key components of the insulin-signaling cascade [33]. 

Evidence suggests that the circulating lipid can cause lipo-toxicity that is associated with the development of IR by activating oxidative stress, inflammation, mitochondrial dysfunction, and cell-death [34]. Oxidative stress can affect the progression of IR via its influence on insulin-stimulated GLUT-4 translocation and glucose uptake through inactivating the PI3K-Akt signaling [35]. We demonstrated that the CLA supplementation significantly suppressed the serum lipid levels and the activities of enzymes related to hepatic function impairment, whereas it increased the activities of hepatic anti-oxidative enzymes in the HF mice. Moreover, we also found that the CLA supplementation significantly improved the serum adiponectin level in the HF mice. Adiponectin has been indicated to ameliorate lipid-induced IR in mice by reducing ectopic lipid storage and promoting the fatty acid oxidation process [36]. In line with this, we demonstrated that the expression of genes related to lipogenesis was significantly decreased, whereas the genes related to fatty acid oxidation were significantly upregulated in the adipose tissue of the HF mice with the CLA supplementation. These findings suggest the significant role of CLA in reversing HF feeding-induced IR by suppressing lipo-toxicity-related signaling and improving anti-oxidative function. 

The liver is a fundamental organ that is critical for metabolism and biosynthesis. The fat accumulation and energy metabolism in the liver also contribute substantially to the development of IR [37]. The H&E staining results confirmed that CLA ameliorated the lipid storage in liver. Simultaneously, hepatic transcriptome results showed that the genes related to metabolism and insulin signaling were differentially expressed between the HF+CLA and HF mice and, thereby, substantiated that CLA administration effectively improved liver function. For example, AMPK signaling, which can act as an energy sensor by promoting fatty acid oxidation and inhibiting gluconeogenesis and fatty acid synthesis [38,39], and which can improve insulin signaling via stimulating the Nrf2 phosphorylation and, subsequently, the activation of GPx4 [40], was significantly enhanced in the HF mice with the CLA supplementation. The metabolic-related signaling pathways such as linoleic acid, arachidonic acid, arginine and proline metabolism were also enhanced in the HF mice with CLA supplemented. Through treating human liver hepatocellular (HepG2) cells with linoleic acid and arachidonic acid, respectively, Mariniello et al. [41] found that these two polyunsaturated fatty acids substantially enhanced the insulin action by increasing the phosphorylation of AKT. Arginine is proposed to be the largest nitrogen-donating amino acid in the body and acts as the precursor of proline. Arginine metabolism has been found to affect multiple processes including amino acid synthesis and the regulation of immune functions, and is also closely related to the progression of T2D [42]. In consistency with these metabolic changes, the insulin and PI3K-Akt signaling pathways were also substantially enhanced in the liver of the HF mice with CLA supplemented. Therefore, the protective effects of CLA in alleviating HF-induced liver dysfunction are largely associated with the regulation of the host metabolism.

Accumulating evidence suggests gut microbiome affects various pathologic conditions including metabolic syndromes like IR and inflammation [43]. We, therefore, investigated the potent involvement of the gut microbiome in mediating the regulation of CLA on dietary HF-induced inflammation, metabolic disorder and IR in the context of microbiota–host signal interactions. The results in the present study demonstrated that the supplementation of CLA significantly improved the abundance of Bacteroidetes, whereas it reduced the abundance of Firmicutes in the HF mice, which was consistent with previous findings that the lean human phenotype was associated with a decreased Firmicutes/Bacteroidetes ratio [44]. Simultaneously, the supplementation of CLA suppressed the gut dysbiosis-related bacteria such as *Streptococcus* and *Anaerotruncus* which are suggested to strongly correlate with the progression of inflammation, IR and metabolic diseases [45,46]. On the other hand, the abundances of potent SCFAs-producing bacteria such as *Muribaculaceae*, *Allobaculum*, *Alloprevotella*, *Alistipes*, *Akkermansia*, *Bacteroides* and *Parabacteroides* as well as the production of specific SCFAs, which have been demonstrated to negatively correlate with the levels of blood glucose and lipid, inflammation and IR, positively correlate with the activities of anti-oxidative enzymes and insulin signaling [47,48,49,50,51,52,53], were both significantly improved with the CLA supplementation in the HF mice. *Akkermansia*, *Bacteroides* and *Parabacteroides* are even regarded as promising probiotics to prevent and treat metabolic syndrome and immune diseases in humans [54]. Studies in humans also indicated that healthy individuals had a significantly higher abundance of butyrate-producing bacteria than T2D patients [55]. Correlation analysis further confirmed the regulatory effects of CLA on alleviating blood glucose and lipid levels, improving glucose tolerance and insulin sensitivity, suppressing lipid accumulation, the expression of signals involved in lipogenesis and inflammation, as well as enhancing hepatic antioxidant capacity and the expression of genes or proteins involved in insulin signaling in adipose tissue or liver were largely derived from these enriched beneficial bacteria that showed close correlation with SCFAs production; also, the specific production of SCFAs. The gut microbiota usually affects the host metabolism via the regulation of its metabolite SCFAs, which can mediate the interaction between the gut and multiple organs by modulating glucose homeostasis, lipid metabolism, fatty acid oxidation, immune responses and insulin signaling, and have been proposed as valuable candidates in the prevention of obesity and T2D [47,56]. For example, treating obese mice with sodium butyrate was indicated to increase insulin sensitivity by enhancing the expression of the insulin receptor in relation to the activation of AKT signaling, as well as alleviating inflammation via downregulating NF-κB signaling in adipose tissue [57]. Treating adipose tissue explanations obtained from overweight patients with propionate was found to attenuate inflammation and enhance glucose uptake effectively [58]. Additionally, SCFAs have been proposed to promote the intestinal hormone secretion such as GLP-1, which can control glucose homeostasis by improving the functions of pancreatic β-cells to regulate insulin secretion as well as inhibiting pancreatic β-cell apoptosis [59,60]. The potential of using the GLP-1 receptor agonist to improve insulin sensitivity in T2D patients has also been previously reported [61]. In the present study, we showed that the CLA administration could effectively increase colonic GLP-1 production in HF mice. Thus, the CLA-induced SCFAs and GLP-1 production may contribute to improved metabolic homeostasis in HF mice. In consideration of the significant role of the diet in shaping gut microbiome, numerous human studies have shown the potential of using therapeutic nutritionally derived strategies to target the gut microbiota and its metabolite with subsequent health benefits to improve metabolic diseases [62]. Therefore, although our study demonstrated the potential of CLA to ameliorate HF-induced IR by modulating microbiota–host metabolic and immunomodulatory interactions in the mouse model, the CLA administration may affect the gut microbiome of humans with IR in a similar way, and future clinical studies should focus on translating these findings in humans to confirm this inference. 

## 5. Conclusions

In conclusion, CLA effectively ameliorated IR and the associated disorders of glycolipid metabolism in HF-fed mice by downregulating the inflammatory responses and improving the insulin signaling in the adipose tissue and liver. Furthermore, CLA dramatically enhanced the abundance of SCFA-producing bacteria as well as SCFAs production in the colon of HF-fed mice, which were demonstrated to closely correlate with the alterations in the expression levels of signals involved in inflammation, glycolipid metabolism and IR. Our findings, thus, showed that CLA was a promising therapeutic candidate to ameliorate IR via regulating microbiota–host metabolic and immunomodulatory interactions.

## Data Availability

The 16S rRNA data obtained in present study are available from the Sequence Read Archive (SRA; https://www.ncbi.nlm.nih.gov/sra) (accessed on 8 March 2023) under the accession number of PRJNA940316. The RNA-Seq data obtained in present study are available from the Sequence Read Archive (SRA; https://www.ncbi.nlm.nih.gov/sra) (accessed on 24 February 2023) under the accession number of PRJNA938011.

## Supplementary Materials

The following supporting information can be downloaded at: https://www.mdpi.com/article/10.3390/nu16081133/s1, Table S1: Primers used in this study; Table S2: Pairwise comparisons on Bray–Curtis of each microbial community; Figure S1: Gene expression profiles among different groups.
