# Supplementary material for: Conjugated Linoleic Acid Ameliorates High Fat-Induced Insulin Resistance via Regulating Gut Microbiota–Host Metabolic and Immunomodulatory Interactions
Source: Nutrients. 2024 Apr 11;16(8):1133. doi: 10.3390/nu16081133 (PMC11053735; doi:10.3390/nu16081133)
Supplement: Supplementary file 1 [file nutrients-16-01133-s001.zip › nutrients-2867005-supplementary.pdf]

nutrients

MDPI

Supplementary Materials

Conjugated Linoleic Acid Ameliorates High Fat-Induced Insulin Resistance via Regulating Gut Microbiota–Host Metabolic and Immunomodulatory Interactions

Linjun Wu [1,†], Shijie Ye [1,†], Xiangfei Deng [1], Zhengwei Fu [1,*], Jinjun Li [2,3,4,5,*] and Chunlei Yang [1,*]

[1] College of Biotechnology and Bioengineering, Zhejiang University of Technology, Hangzhou 310014, China; 2112005190@zjut.edu.cn (L.W.); yeshijie0423@163.com (S.Y.); 2112005101@zjut.edu.cn (X.D.)
[2] Food Science Institute, Zhejiang Academy of Agricultural Sciences, Hangzhou 310021, China
[3] Key Laboratory of Postharvest Preservation and Processing of Vegetables (Co-Construction by Ministry and Province), Ministry of Agriculture and Rural Affairs, Hangzhou 310021, China
[4] Key Laboratory of Post-Harvest Handling of Fruits, Ministry of Agriculture and Rural Affairs, Hangzhou 310021, China
[5] Key Laboratory of Fruits and Vegetables Postharvest and Processing Technology Research of Zhejiang Province, Hangzhou 310021, China
* Correspondence: azwfu@zjut.edu.cn (Z.F.); lijinjun@zaas.ac.cn (J.L.); chunleiyang@zjut.edu.cn (C.Y.)
† These authors contributed equally to this work.

Table S1 Primers used in this study.

Name	Primer（5′-3′）
TNF-α	Forward: GCTTGTCACTCGAATTTTGAGA
	Reverse: CCTCTGGCGAATGGCTTTAC
IL-1β	Forward: GCAACTGTTCCTGAACTCAACT
	Reverse: ATCTTTTGGGGTCCGTCAACT
IL-6	Forward: TAGTCCTTCCTACCCCAATTTCC
	Reverse: TTGGTCCTTAGCCACTCCTTC
IL-10	Forward: GCTCTTACTGACTGGCATGAG
	Reverse: CGCAGCTCTAGGAGCATGTG
CD36	Forward: ATGGGCTGTGATCGGAACTG
	Reverse: GTCTTCCCAATAAGCATGTCTCC
FABP1	Forward: TGGTCCGCAATGAGTTCACCCT
	Reverse: CCAGCTTGACGACTGCCTTGACTT
SLC27A1	Forward: CGCTTTCTGCGTATCGTCTG
	Reverse: GATGCACGGGATCGTGTCT
SREBP1	Forward: CACTTCTGGAGACATCGCAAAC
	Reverse: ATGGTAGACAACAGCCGCATC
ACC	Forward: AGGATTTGCTGTTTCTCAGAGCTT
	Reverse: CAGGATCTACCCAGGCCACAT
PPARα	Forward: AGACAAAGAGGCAGAGGTCC
	Reverse: AAGGAGGACAGCATCGTGAA
CPT1	Forward: AGGACCCTGAGGCATCTATT
	Reverse: ATGACCTCCTGGCATTCTCC
PGC1α	Forward: CCGTCTCTACTTAAGAAGCTC
	Reverse: GTTCTGAGTGCTAAGACCG
β-actin	Forward: GGCTGTATTCCCCTCCATCG
	Reverse: CCAGTTGGTAACAATGCCATGT

Table S2 Pairwise comparisons on Bray-Curtis of each microbial community.

p-value[1]	NC	NC+CLA[3]	HF+CLA
NC[2]	-	0.065	0.009**
HF[4]	0.009**	0.009**	0.049*
HF+CLA[5]	0.009**	0.005**	-

[1] $^{*}p < 0.05$, $^{**}p < 0.01$ according to Bray-Curtis using ANOSIM.

[2] NC, mice fed control diet for 15 weeks ($n = 6$).

[3] NC+CLA, mice fed control diet with 400 mg/kg body weight CLA supplemented every day for 15 weeks ($n = 6$).

[4] HF, mice fed HF diet for 15 weeks ($n = 6$).

[5] HF+CLA, mice fed HF diet with 400 mg/kg body weight CLA supplemented every day for 15 weeks ($n = 6$).

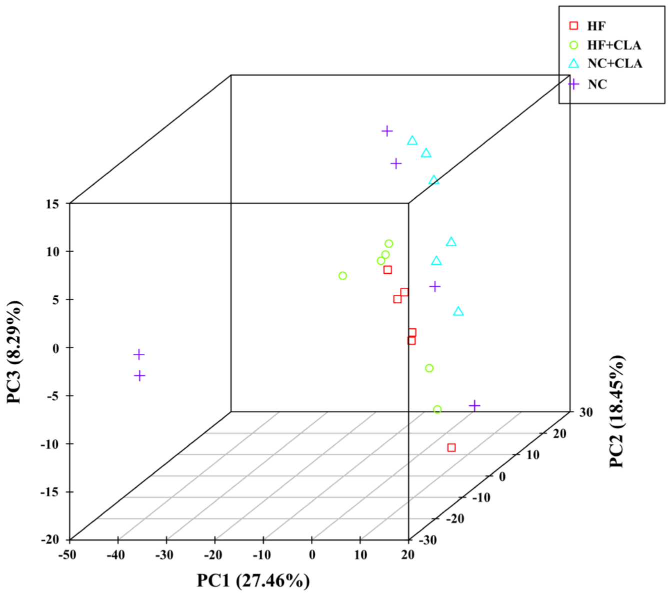

Figure S1. Gene expression profiles among different groups. Principal component analysis (PCA) profile of the gene expression in the liver of mice among NC, NC+CLA, HF and HF+CLA groups. NC, mice fed control diet for 15 weeks ($n = 6$); NC+CLA, mice fed control diet with 400 mg/kg body weight CLA supplemented every day for 15 weeks ($n = 6$); HF, mice fed HF diet for 15 weeks ($n = 6$); HF+CLA, mice fed HF diet with 400 mg/kg body weight CLA supplemented every day for 15 weeks ($n = 6$).
